# Underexpression of mitochondrial-DNA encoded ATP synthesis-related genes and DNA repair genes in systemic lupus erythematosus

**DOI:** 10.1186/ar3317

**Published:** 2011-04-15

**Authors:** Hooi-Ming Lee, Hidehiko Sugino, Chieko Aoki, Norihiro Nishimoto

**Affiliations:** 1 Graduate School of Frontier Biosciences, Osaka University, 1-3 Yamada-Oka, Suita, Osaka 565-0871, Japan; 2 Laboratory of Immune Regulation, Wakayama Medical University, 105 Saito Bio Innovation Center, 7-7-20 Saito-Asagi, Ibaraki, Osaka 567-0085, Japan

## Abstract

**Introduction:**

Systemic lupus erythematosus (SLE) is a prototypical autoimmune disease characterized by various systemic symptoms and multiple organ damage. We clarify biological and functional abnormalities in SLE by comparing the gene expression profiles of SLE patients with those of healthy individuals.

**Methods:**

Gene expression profiles from the peripheral blood of 21 SLE patients and 45 healthy individuals were obtained using a DNA microarray. Gene ontology analysis and network pathway analysis were performed on the genes differentially expressed between SLE and healthy individuals.

**Results:**

A total of 2,329 upregulated genes and 1,884 downregulated genes were differentially expressed. Gene ontology analysis revealed that the upregulated genes were classified as response to biotic stimulus genes, which mainly includes genes related to immune response. Abnormalities in other categories such as cell motility and regulation of apoptosis were also revealed. Downregulated genes were mainly sorted into two gene categories, sensory perception and response to radiation/light. The sensory perception genes included ATPase/ATPase domain-containing genes, myosin-related genes, and two excision repair cross-complementing genes, which are involved in DNA repair. Other genes in this group - including three crystallin genes, genes encoding the receptor protein for melanocyte-stimulating hormone, and six mitochondrial-DNA encoded genes, which are involved in ATP synthesis - were also categorized as response to radiation genes. Using network pathway analysis, IL-6, transforming growth factor beta 1, TNF, and hepatocyte nuclear factor 4α were found to play central roles in the networks of sensory perception-related molecules.

**Conclusions:**

Functional abnormalities in ATP synthesis and DNA repair are implicated in peripheral blood cells from SLE patients.

## Introduction

Systemic lupus erythematosus (SLE) is a prototypical autoimmune disease characterized by various clinical manifestations, high titers of autoantibodies, and multiple organ damage [1]. Multiple genetic and environmental factors are thought to influence the disease progress, but details of the mechanisms of SLE clinical manifestations or the biological processes behind them remain obscure. The role of environmental factors pathologically involved in SLE, especially regarding skin lesions after sun exposure, has been reported [2]. In addition, abnormalities in apoptosis, impaired clearance of dying cells, hyper-reactive B cells and T cells in the immune system, and many other SLE pathophysiologies have also been investigated [3,4]. From an autoimmunologic viewpoint, disruption of self-tolerance is implicated through distortion in the cell-cell communications and cytokine networks. Nevertheless, there are few reports comprehensively considering the environmental factors in combination with aberrant biological or cellular functions in SLE, which involve a substantial number of molecules.

DNA microarrays can be amenable to exhaustively analyze the gene expressions of such multiple molecules. Indeed, Bennett and colleagues have demonstrated using a microarray that type I interferon and its related molecules as well as granulopoiesis-related molecules play central roles in SLE [5]. We and other researchers, also using microarray analysis, confirmed the interferon signature in peripheral blood cells from patients with SLE [6-8], where IFNα, IFNβ, and TNF may interact with each other in regulating the immune response molecules [8,9]. Despite these important findings in immune response, because SLE is a systemic disease that influences multiple organs, it is also important to clarify other biological or cellular functional abnormalities relevant to SLE clinical manifestations other than immunological response abnormality. In the present study, we attempt to identify such abnormalities using differentially expressed genes exhaustively analyzed by DNA microarray together with bioinformatics analysis.

## Materials and methods

### Patients and healthy individuals

Twenty-one patients (all women, median age 35 years, range 26 to 72 years) with SLE according to the diagnostic criteria of the American College of Rheumatology [10] and 45 healthy individuals (23 males, 22 females) were enrolled in the present study after providing written informed consent. The study was approved by the Ethical Committee of Wakayama Medical University for clinical studies on human subjects. Twenty SLE patients were treated with prednisolone <20 mg/day, and the remaining one patient at 20 mg/day. Three of these 21 patients were treated with cyclosporine, with azathioprine, or with methotrexate in combination with prednisolone, respectively.

The median disease activity of SLE patients based on the SLE Disease Activity Index (SLEDAI) 2000 score was 6 (range 2 to 24) [11]. One patient was in a very active state (SLEDAI 2000 score >12), 17 patients were in active states (SLEDAI 2000 score = 4 to 12), and the remaining three patients were not active (SLEDAI 2000 score <4). The median of the assessment based on the BILAG index was 3 (range 1 to 13) [12]. Meanwhile, the median of total white blood cells from SLE patients was 6,150/mm^3 ^(range 2,900 to 12,230/mm^3^). The median for the total number of and the proportion of neutrophils was 4,928/mm^3 ^and 80.0%, respectively (range 1,601 to 9,674/mm^3 ^and 55.2 to 90.1%), while that for lymphocytes was 919/mm^3 ^and 14.7%, respectively (range 376 to 1517/mm^3 ^and 4.7 to 24.5%).

### DNA microarray and data analysis

Peripheral blood was collected directly into PAXGene^® ^tubes (Qiagen, Valencia, CA, USA). Total RNA was extracted using the PAXGene Blood RNA kit^® ^(Qiagen) with optimal on-column DNase digestion. Amino allyl RNA (aRNA) was synthesized from 1 μg total RNA using the Amino Allyl MessageAmp™ aRNA kit (Ambion, Austin, TX, USA). Five micrograms of aRNA from each sample (21 SLE patients and 45 healthy individuals) and the equivalent quantity of reference aRNA from a mixture of RNA extracted from peripheral blood of 45 healthy individuals were subjected to Cy3 and Cy5 labeling, respectively. Both labeled aRNA samples were mixed in equal amounts and hybridized with an oligonucleotide-based DNA microarray, AceGene^® ^(HumanOligoChip30K; DNA Chip Research, Yokohama, Japan), which contained 30,000 human genes. The microarrays were scanned using ScanArray Lite^® ^(PerkinElmer, Boston, MA, USA).

Signal values were calculated using DNASIS Array^® ^(Hitachi Software Engineering, Tokyo, Japan) according to the manufacturer's instructions. The intensities of no-probe spots were used as the background. The median and standard deviation of background levels were calculated. Genes whose intensities were less than the median plus two standard deviations of background level were identified as null. The Cy3/Cy5 ratios of all spots on the DNA microarray were normalized by the global ratio median. Only gene expression data that were collected from at least 80% of samples from each group were selected for further analysis. Microarray data have been deposited in the NCBI Gene Expression Omnibus [GEO:GSE17755].

### Gene ontology and network pathway analysis

Genes identified to be differentially expressed according to microarray analysis between SLE patients and healthy individuals were functionally categorized using Expression Analysis Systematic Explorer (EASE) version 2.0 bioinformatics software [13]. Interactions among the molecules of which the genes were differentially expressed in their respective gene categories were investigated using Ingenuity Pathway Analysis version 8.0 [14]. Networks generated by less than five uploaded genes were excluded from the analysis.

### Statistical analysis

The unpaired Mann-Whitney test was used to determine statistically significant differences in the mRNA expression levels between the SLE and healthy groups. Correlation was measured using Spearman's rank correlation. The criterion for statistical significance was *P *< 0.05.

## Results

### Gene ontology analysis on the differentially expressed genes compared between SLE patients and healthy individuals

DNA microarray analysis revealed that 4,213 genes were differentially expressed in peripheral blood cells from patients with SLE compared with healthy individuals: 2,329 out of the 4,213 genes were upregulated, while the remaining 1,884 genes were downregulated. Such a large number of the differentially expressed genes appeared to reflect the pathological complexity involving many molecules of this systemic disease with various clinical manifestations.

To identify any aberrant biological functions in the peripheral blood cells of SLE patients, we performed EASE analysis based on the Gene Ontology database, which can classify a large list of genes into functionally related gene groups and rank the importance of these functional groups on the differentially expressed genes. EASE analysis classifies the gene groups into three Gene Ontology systems: biological process, cellular component, and molecular function. EASE results for the biological process system for upregulated and downregulated genes are shown in Tables 1 and 2, respectively. The EASE score, which is a modified Fisher exact test, represents the probability that over-representation of a certain gene category occurs by chance. Based on common genes, gene categories were further divided into subsets. Each subset of the gene categories was then ordered hierarchically based on the gene list. Identical gene lists are listed as one gene category. The list parameter refers to the total number of upregulated/downregulated genes annotated in the Gene Ontology system (data not shown). There were in total 1,759 genes in the list for 2,329 upregulated genes and 1,429 genes in the list for 1,884 downregulated genes. List hits represent the number of upregulated/downregulated genes that belong to the respective gene category. The population parameter reports all genes annotated in the Gene Ontology system (data not shown). The total number of genes in the population of the biological process system was 13,802. Population hits show the number of genes that belong to the respective gene category in the system.

**Table 1:** Deviated GO Biological Process gene categories of upregulated genes in peripheral blood of SLE patients

Gene category	List hits (*n *= 1,759)	Population hits (*n *= 13,802)	EASE score (<0.01)
Response to external stimulus	265	1,539	6.56 × 10^-8^
Response to biotic stimulus	192	963	7.36 × 10^-11^
Defense response	176	887	7.69 × 10^-10^
Immune response	159	792	2.45 × 10^-9^
Response to stress	147	872	2.39 × 10^-4^
Response to pest/pathogen/parasite	97	501	2.09 × 10^-5^
Response to wounding	59	303	8.83 × 10^-4^
Inflammatory response	48	187	2.68 × 10^-6^
Cell growth and/or maintenance	572	4,092	3.09 × 10^-3^
Modification-dependent protein catabolism	28	123	2.99 × 10^-3^
Ubiquitin-dependent protein catabolism	27	122	5.34 × 10^-3^
Cell motility	62	342	3.78 × 10^-3^
Regulation of apoptosis	42	214	4.60 × 10^-3^
Induction of apoptosis by extracellular signals	11	35	9.91 × 10^-3^

Deviated Gene Ontology database (GO) Biological Process gene categories of upregulated genes in peripheral blood of systemic lupus erythematosus (SLE) patients compared with healthy individuals. EASE, Expression Analysis Systematic Explorer software, version 2.0.

**Table 2:** Deviated GO Biological Process gene categories of downregulated genes in peripheral blood of SLE patients

Gene category	List hits (*n *= 1,429)	Population hits (*n *= 13,802)	EASE score (<0.01)
Sensory perception	56	383	7.49 × 10^-3^
Perception of sound	21	82	2.00 × 10^-4^
Hearing	20	81	4.86 × 10^-4^
Response to radiation	36	222	7.48 × 10^-3^
Response to light	35	207	4.39 × 10^-3^
Calcium ion transport	16	66	2.57 × 10^-3^
Macromolecule biosynthesis	131	1,002	3.38 × 10^-3^
Protein biosynthesis	90	650	3.38 × 10^-3^

Deviated Gene Ontology database (GO) Biological Process gene categories of downregulated genes in peripheral blood of systemic lupus erythematosus (SLE) patients compared with healthy individuals. EASE, Expression Analysis Systematic Explorer software, version 2.0.

EASE analysis of the upregulated genes identified four major gene categories: response to external stimulus, cell growth and/or maintenance, cell motility, and regulation of apoptosis (Table 1). The top-three most-significant categories based on the EASE score - which include response to biotic stimulus, defense response, and immune response - were grouped into the response to external stimulus category and were arranged hierarchically. The gene category cell growth and/or maintenance included ubiquitin-dependent protein catabolism genes. Finally, 62 upregulated genes belonged to the category cell motility, in which 22 of the genes related to inflammatory response or antigen presentation (data not shown) while 42 upregulated genes were in the category regulation of apoptosis.

On the other hand, EASE analysis of the downregulated genes identified four major gene categories: sensory perception, response to radiation, calcium ion transport, and macromolecule biosynthesis (Table 2). The sensory perception category included ATPase/ATPase domain-containing genes and two excision repair cross-complementing genes (ERCC2, ERCC5). Six mitochondrial DNA (mtDNA)-encoded genes - including ATP synthase 6 (ATP6), cytochrome c oxidase (COX)1, COX3, cytochrome b (CYTB), NADH dehydrogenase subunit (ND)1, and ND2 - three crystallin genes, and genes encoding the receptor protein for melanocyte-stimulating hormone (melanocortin 1 receptor) were grouped into both the sensory perception category and the response to radiation category. Except for melanocortin 1 receptor, the 36 genes in the response to radiation category were also in the response to light category. These results suggested the possible existence of abnormalities in the above categorized functions.

### Upregulated genes in the category regulation of apoptosis and their network pathway analysis

**Figure 1:**
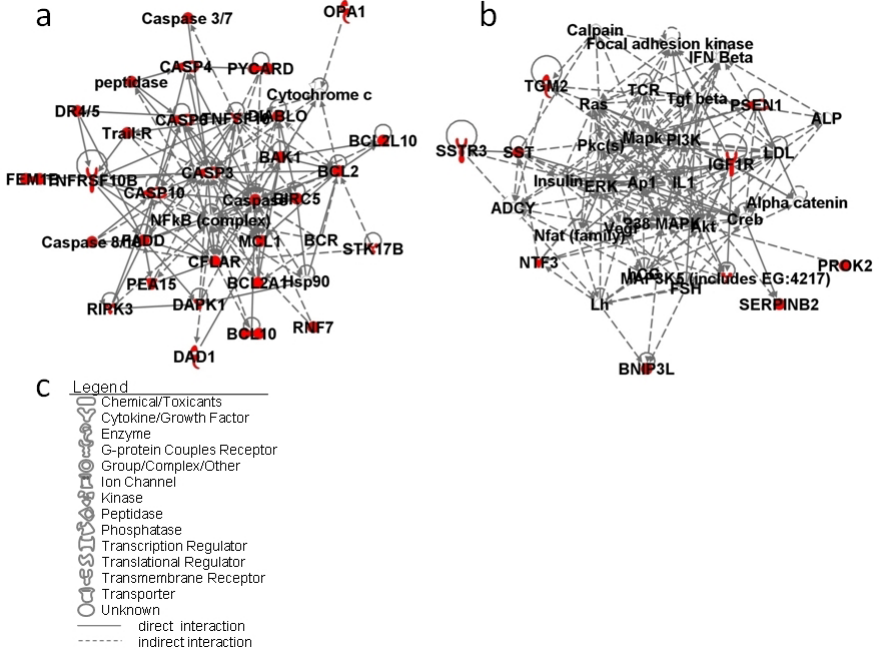
**Network pathway analysis of upregulated genes in the gene category regulation of apoptosis**. **(a) **Network 1 and **(b) **Network 2 constructed by 42 upregulated genes. **(c) **Network graphical representation. Genes or gene products are represented as individual nodes whose shapes represent the functional class of the gene products. The biological relationship between the two nodes is represented as an edge (line). All edges are supported by at least one reference from the literature stored in the Ingenuity Pathways Knowledge Base (IPKB). Genes with colored nodes were found over-represented in the gene category regulation of apoptosis. Genes with uncolored nodes were not found over-represented, but were depicted by computationally generated networks on the basis of evidence stored in the IPKB, indicating strong biologic relevance to that network.

Here, we focus on the 42 upregulated genes in the category regulation of apoptosis. In order to identify the relationship among these molecules and the centered molecules in the networks, a network-based analysis was conducted on these molecules. Two networks were represented by the 42 upregulated genes (Figure 1).

Twenty-five of the 42 genes created the first network (Figure 1a) with the NF-κB complex and caspase complex at the center. Caspases, or cysteine-aspartic proteases, are a family of cysteine proteases that play essential roles in apoptosis, necrosis and inflammation. Caspase (CASP)3, CASP4, CASP6, and CASP10 genes are in this network. Also present in the first network are the CASP8 and FADD-like apoptosis regulator genes, the B-cell CLL/lymphoma 2 gene, and TNF superfamily-related genes, all of which are involved in the caspase cascade, and the optic atrophy1 (OPA1) gene. OPA1 is a component of the mitochondrial network and is involved in the positive regulation of anti-apoptosis.

Meanwhile, many transcription factors such as p38 mitogen-activated protein kinase, extracellular signal-regulated kinase, Ap1, and Akt represent the center of the second network (Figure 1b). Presenilin1, which relates to mitochondrial dysfunction, is also depicted in this network.

### Downregulated genes in the category sensory perception and their network pathway analysis

**Figure 2:**
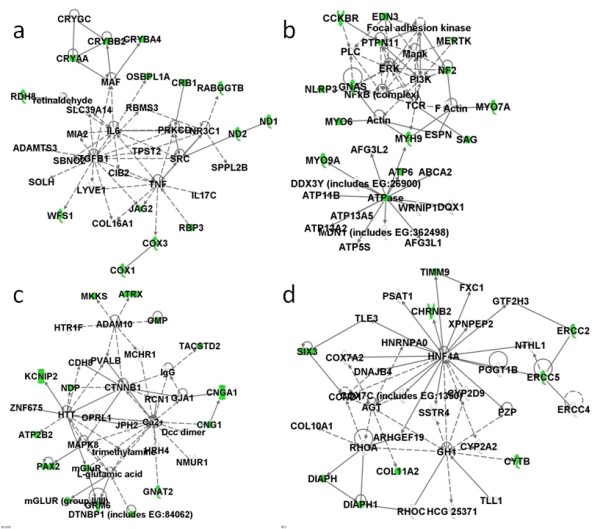
**Network pathway analysis of downregulated genes in the gene category sensory perception**. **(a) **Network 1, **(b) **Network 2, **(c) **Network 3, and **(d) **Network 4 constructed by 56 downregulated genes.

The downregulated genes categorized into sensory perception included ATPase/ATPase domain-containing genes, two ERCC genes (ERCC2 and ERCC5), as well as six mtDNA-encoded genes. Using network pathway analysis on the 56 genes in sensory perception, four networks were constructed (Figure 2).

A cluster of crystallin genes, COX1, COX3, ND1, and ND2 are represented in the first network, in which IL-6, transforming growth factor beta 1, and TNF are at the center (Figure 2a). The second network has extracellular signal-regulated kinase, NF-κB, and mitogen-activated protein kinase at the center (Figure 2b). The calcium ion plays central roles in the third network (Figure 2c), while hepatocyte nuclear factor 4α is important in the final network (Figure 2d). ERCC2, ERCC5, and CYTB were also found in the last network. The results also showed that, among the 56 downregulated molecules, there are six molecules involved in the pathways of oxidative phosphorylation (ATP6, COX1, COX3, CYTB, ND1, and ND2), in which three are relevant to mitochondrial dysfunction.

**Figure 3:**
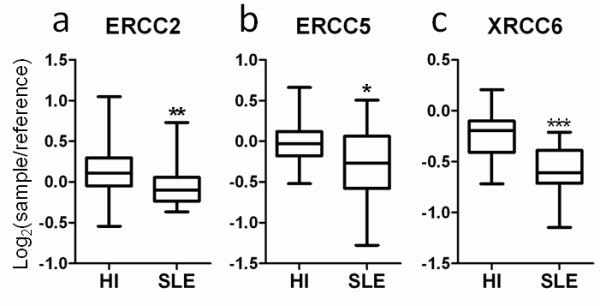
**Decrease in the expressions of three DNA repair genes**. The expression levels of **(a) **excision repair cross-complementing (ERCC)2, **(b) **ERCC5, and **(c) **X-ray repair cross-complementing (XRCC)6 in peripheral blood of 21 patients with systemic lupus erythematosus (SLE) and 45 healthy individuals (HI) are shown. All data represent microarray data with the expression values of log_2_(sample/reference). *P < 0.05, **P < 0.01, ***P < 0.001 (unpaired Mann-Whitney test) for 21 SLE patients versus 45 HI. Boxes contain the 50% of values falling between the 25th and 75th percentiles, the horizontal line within the box represents the median value, and the whiskers are the lines that extended from the box to the highest and lowest values, excluding outliers.

**Figure 4:**
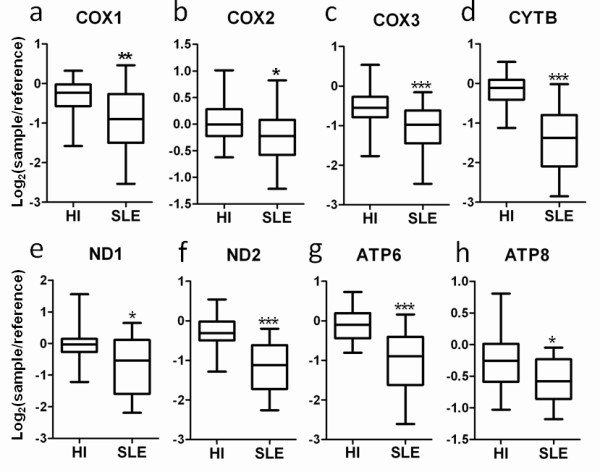
**Decrease in the expressions of nine mitochondrial DNA-encoded genes**. The expression levels of **(a) **cytochrome c oxidase (COX)1, **(b) **COX2, **(c) **COX3, **(d) **cytochrome b (CYTB), **(e) **NADH dehydrogenase subunit (ND)1, **(f) **ND2, **(g) **ATP synthase (ATP)6, and **(h) **ATP8 in peripheral blood of 21 patients with systemic lupus erythematosus (SLE) and 45 healthy individuals (HI) are shown. All data represent microarray data with the expression values of log_2_(sample/reference). *P < 0.05, **P < 0.01, ***P < 0.001 (unpaired Mann-Whitney test) for 21 SLE patients versus 45 HI. Boxes contain the 50% of values falling between the 25th and 75th percentiles, the horizontal line within the box represents the median value, and the whiskers are the lines that extended from the box to the highest and lowest values, excluding outliers.

Because the expressions of DNA repair and mtDNA-encoded genes were found downregulated, the relevant gene expression levels were further investigated. Besides the molecules mentioned above (ERCC2, ERCC5, CYTB, COX1, COX3, ND1, ND2, and ATP6), the expression levels of X-ray repair cross-complementing 6, COX2, and ATP8 were also decreased in patients with SLE (Figures 3 and 4). Several ATPases and ATP synthases such as ATP2B1, ATP2B2, ATP5D, ATP5S, ATP6V1H, ATP8A2, and ATP10B were also significantly downregulated (unpaired Mann-Whitney test, *P *< 0.01) (data not shown).

On the other hand, among the 21 SLE patients eight had a history for manifestations of photosensitivity. Among the 11 molecules identified in Figures 3 and 4, only X-ray repair cross-complementing 6 expressions correlated with the SLEDAI from the eight patients. Meanwhile, we found only ND1 and ND2 expressions correlated with the SLEDAI of all 21 SLE patients (data not shown). These results may in part be due to the small range of gene expression-level data gathered from the AceGene^® ^microarray and the small variability in SLEDAI of the patients recruited in the present study.

## Discussion

In the present study, we compared the gene expression profiles of 21 SLE patients (all females) with those of 45 healthy controls consisting of 23 males and 22 females. Since all SLE patients are female, we also limited our comparison to the gene expression profiles of the 21 SLE patients with the 22 female controls only, finding no changes to our conclusions. We include the 23 males so that we can use the same control group for later studies when comparing other disease controls.

We and other researchers have reported that NF-κB signaling pathways play significant roles in the aberrant immunoregulatory networks of SLE and other autoimmune disorders [8,15,16]. NF-κB is a key transcription factor that regulates the expression of a wide range of genes, including those involved in immune response, cell adhesion, differentiation, proliferation, and apoptosis. Notably, Oikonomidou and colleagues demonstrated that impaired NF-κB signaling observed in SLE patients can be partially explained by a decrease in NF-κB binding to DNA [17]. In the present study, we found aberrant expression of the genes relevant to the categories regulation of apoptosis and response to light. Since the NF-κB complex was depicted in the center of these genes by network pathway analysis, NF-κB has again been thought to play a pathological role in regulating apoptosis and response to light.

ERCC2, also called xeroderma pigmentosum complementation group D (XPD), encodes a protein involved in transcription-coupled nucleotide excision repair. Defects in the ERCC2 gene can result in three different disorders: the cancer-prone XPD syndrome, trichothiodystrophy, and aging disorders Cockayne syndrome, which is characterized by severe growth defects, mental retardation, and cachexia [18,19]. ERCC2 is also a part of human transcriptional initiation factor TFIIH and has ATP-dependent helicase activity. ERCC5, also called xeroderma pigmentosum complementation group G (XPG), also encodes a DNA repair protein. ERCC5 is involved in excision repair of UV-induced DNA damage. Mutations of this gene cause XPG syndrome or Cockayne syndrome. Worth noting is the fact that the ERCC2/XPD and ERCC5/XPG proteins are both involved in excision repair of UV-induced DNA damage and that photosensitivity is commonly observed in patients with xeroderma pigmentosum, Cockayne syndrome, and trichothiodystrophy.

Since enhanced photosensitivity is also a common clinical symptom for SLE, an abnormal expression of ERCC2/XPD and ERCC5/XPG may be pathologically involved in photosensitivity of SLE. An analysis of ERCC2/XPD polymorphisms in patients with SLE showed that these had no association with genetic susceptibility in SLE [20]. Bassi and colleagues, however, reported that SLE leucocytes less efficiently repair radiation-induced DNA damage and that DNA repair gene polymorphic sites may predispose to the development of particular clinical and laboratory features such as neuropsychiatric manifestations and antiphospholipid antibody syndrome, although a significant association was not observed in SLE patients [21]. Here we found ERCC genes in the peripheral blood cells of SLE patients to be underexpressed. It is important to confirm the gene expression levels of DNA repair genes in skin, as skin is the outer surface organ that directly comes into contact with the environment, as is the case for sun exposure. Although the detailed biological characteristics of photosensitivity are still unknown, it is possible that UV light induces the accumulation of damaged DNA due to decrease in ERCC expression. As a result, abnormal apoptosis can occur, which results in poor disposal of cell debris including DNA. This in turn can lead to an overexpression of interferons by antigen-presenting cells, including plasmacytoid dendritic cells, which ultimately can lead to SLE systemic symptoms. ERCCs are also involved in the transcription-coupled repair of oxidative DNA lesions. A decrease in ERCC expression may thus contribute the susceptibility to oxidative stress in SLE.

Abnormalities in the mitochondria have been a topic of interest for SLE studies for several years [22]. It has been reported that lupus T cells exhibit mitochondrial hyperpolarization, resulting in ATP depletion, and thus contribute to abnormal T-cell activation and cell death in patients with SLE [23]. We report that certain gene groups related to the function of sensory perception are underexpressed in the peripheral blood cells of SLE patients. Notably, of the relevant genes, six are mtDNA-encoded genes (ATP6, COX1, COX3, CYTB, ND1, and ND2) that also function in oxidative phosphorylation, where defects in three of them (COX1, COX3, and CYTB) also lead to mitochondrial dysfunction.

Also worth noting is that repairs of UV-induced DNA damage by ERCC2/XPD and ERCC5/XPG requires ATP. The ATP and DNA binding regions are contained in ERCC2/XPD [24]. Most amino-acid substitution variants of ERCC2/XPD found in patients with xeroderma pigmentosum, Cockayne syndrome, and trichothiodystrophy occur in these regions. Although the reports mentioned above found no genetic variations in ERCC2/XPD or ERCC5/XPG when they were linked to SLE, the observed mitochondrial dysfunction in SLE, which implicates ATP depletion [20,21], combined with the underexpression of ATP-dependent ERCC genes suggests impaired DNA repair or consequently increased apoptosis, both of which may contribute the clinical or laboratory manifestations of SLE. Moreover, OPA1 and nuclear respiratory factor 1 expressions were found to increase (data not shown). OPA1 is necessary for the synthesis of new mitochondrial components, while nuclear respiratory factor 1 functions as a transcription factor that activates the expression of certain nuclear genes required for mtDNA transcription and replication [25,26]. Perl's group has added that persistent mitochondrial hyperpolarization is associated with increased mitochondrial biogenesis in SLE T cells [27], although the molecules above were not mentioned in their study. The increase in OPA1 and nuclear respiratory factor 1 expressions may compensate for the mitochondrial dysfunction seen in SLE.

In our previous study in systemic juvenile idiopathic arthritis, we showed abnormal downregulation of genes related to oxidative phosphorylation, suggesting a mitochondrial disorder [28]. It is interesting that we also identified downregulation of mtDNA-encoded genes involved in oxidative phosphorylation in the present study. Slight downregulation of ERCC2 expression was also observed in systemic juvenile idiopathic arthritis. We did not, however, identify other abnormal expressions of ERCC genes or oxidative phosphorylation-related genes in rheumatoid arthritis or polyarticular type juvenile idiopathic arthritis, although a downregulation of the gene expressions for ATP6 and CYTB were found in rheumatoid arthritis (unpublished data).

Moreover, it is interesting that proinflammatory cytokines such as IL-6 and TNF, as well as anti-inflammatory cytokines including transforming growth factor beta, were found to play central roles in the networks of sensory perception molecules, which included oxidative phosphorylation-related molecules. The roles of cytokines in the immunoregulatory network of autoimmune diseases as well as the relationship of mitochondria with apoptosis have been previously reported, while few studies have described the relationships between cytokines and mitochondria [29-33]. These reports suggested that we cannot eliminate the possibility that chronic inflammation with imbalanced cytokine homeostasis may alter mitochondrial function.

## Conclusions

Functional abnormalities of ATP synthesis and DNA repair were implicated in peripheral blood cells from patients with SLE, but more investigation needs to be conducted to further elucidate the mechanisms involved in SLE.

## Abbreviations

aRNA: amino allyl RNA; ATP6: ATP synthase 6; CASP: caspase; COX: cytochrome c oxidase; CYTB: cytochrome b; EASE: Expression Analysis Systematic Explorer; ERCC: excision repair cross-complementing; IFN: interferon; IL: interleukin; mtDNA: mitochondrial DNA; ND: NADH dehydrogenase subunit; NF-κB: nuclear factor of kappa light polypeptide; OPA1: optic atrophy 1; SLE: systemic lupus erythematosus; SLEDAI: SLE Disease Activity Index; TNF: tumor necrosis factor; XPD: xeroderma pigmentosum complementation group D; XPG: xeroderma pigmentosum complementation group G.

## Competing interests

The authors declare that they have no competing interests.

## Authors' contributions

H-ML performed the statistical analysis and interpretation of the microarray studies, and was involved in drafting the manuscript or revising it critically for important intellectual content. HS assisted with data analysis. CA performed labeling and scanning of the microarrays. NN made substantial contributions to the conception and design or analysis and interpretation of data. All authors read and approved the final manuscript.

## References

[B1] Kotzin BL Systemic lupus erythematosus Cell 1996 85 303 306 10.1016/S0092-8674(00)81108-3 8616885

[B2] Lehmann P Homey B Clinic and pathophysiology of photosensitivity in lupus erythematosus Autoimmun Rev 2009 8 456 461 10.1016/j.autrev.2008.12.012 19167524

[B3] Nagata S Autoimmune diseases caused by defects in clearing dead cells and nuclei expelled from erythroid precursors Immunol Rev 2007 220 237 250 10.1111/j.1600-065X.2007.00571.x 17979851

[B4] Peng SL Altered T and B lymphocyte signaling pathways in lupus Autoimmun Rev 2009 8 179 183 10.1016/j.autrev.2008.07.040 18721908

[B5] Bennett L Palucka AK Arce E Cantrell V Borvak J Banchereau J Pascual V Interferon and granulopoiesis signatures in systemic lupus erythematosus blood J Exp Med 2003 197 711 723 10.1084/jem.20021553 12642603 PMC2193846

[B6] Baechler EC Batliwalla FM Karypis G Gaffney PM Ortmann WA Espe KJ Shark KB Grande WJ Hughes KM Kapur V Gregersen PK Behrens TW Interferon-inducible gene expression signature in peripheral blood cells of patients with severe lupus Proc Natl Acad Sci USA 2003 100 2610 2615 10.1073/pnas.0337679100 12604793 PMC151388

[B7] Feng X Wu H Grossman JM Hanvivadhanakul P FitzGerald JD Park GS Dong X Chen W Kim MH Weng HH Furst DE Gorn A McMahon M Taylor M Brahn E Hahn BH Tsao BP Association of increased interferon-inducible gene expression with disease activity and lupus nephritis in patients with systemic lupus erythematosus Arthritis Rheum 2006 54 2951 2962 10.1002/art.22044 16947629

[B8] Lee HM Mima T Sugino H Aoki C Adachi Y Yoshio-Hoshino N Matsubara K Nishimoto N Interactions among type I and type II interferon, tumor necrosis factor, and beta-estradiol in the regulation of immune response-related gene expressions in systemic lupus erythematosus Arthritis Res Ther 2009 11 R1 10.1186/ar2584 19121222 PMC2688231

[B9] Palucka AK Blanck JP Bennett L Pascual V Banchereau J Cross-regulation of TNF and IFN-α in autoimmune diseases Proc Natl Acad Sci USA 2005 102 3372 3377 10.1073/pnas.0408506102 15728381 PMC552921

[B10] Tan EM Cohen AS Fries JF Masi AT McShane DJ Rothfield NF Schaller JG Talal N Winchester RJ The 1982 revised criteria for the classification of systemic lupus erythematosus Arthritis Rheum 1982 25 1271 1277 10.1002/art.1780251101 7138600

[B11] Gladman DD Ibanez D Urowitz MB Systemic lupus erythematosus disease activity index 2000 J Rheumatol 2002 29 288 291 11838846

[B12] Hay EM Bacon PA Gordon C Isenberg DA Maddison P Snaith ML Symmons DP Viner N Zoma A The BILAG index: a reliable and valid instrument for measuring clinical disease activity in systemic lupus erythematosus Q J Med 1993 86 447 458 8210301

[B13] EASE: the Expression Analysis Systematic Explorer http://david.abcc.ncifcrf.gov/ease/ease.jsp

[B14] Ingenuity Systems http://www.ingenuity.com

[B15] Brown KD Claudio E Siebenlist U The roles of the classical and alternative nuclear factor-κB pathways: potential implications for autoimmunity and rheumatoid arthritis Arthritis Res Ther 2008 10 212 10.1186/ar2457 18771589 PMC2575629

[B16] Kurylowicz A Nauman J The role of nuclear factor-κB in the development of autoimmune diseases: a link between genes and environment Acta Biochim Pol 2008 55 629 647 19081854

[B17] Oikonomidou O Vlachoyiannopoulos PG Kominakis A Kalofoutis A Moutsopoulos HM Moutsatsou P Glucocorticoid receptor, nuclear factor κB, activator protein-1 and C-jun N-terminal kinase in systemic lupus erythematosus patients Neuroimmunomodulation 2006 13 194 204 10.1159/000100474 17347585

[B18] Lehmann AR The xeroderma pigmentosum group D (XPD) gene: one gene, two functions, three diseases Genes Dev 2001 15 15 23 10.1101/gad.859501 11156600

[B19] Clarkson SG The XPG story Biochimie 2003 85 1113 1121 10.1016/j.biochi.2003.10.014 14726017

[B20] Wan L Lin YJ Sheu JJ Huang CM Tsai Y Tsai CH Wong W Tsai FJ Analysis of ERCC2/XPD functional polymorphisms in systemic lupus erythematosus Int J Immunogenet 2009 36 33 37 10.1111/j.1744-313X.2008.00817.x 19055600

[B21] Bassi C Xavier D Palomino G Nicolucci P Soares C Sakamoto-Hojo E Donadi E Efficiency of the DNA repair and polymorphisms of the XRCC1, XRCC3 and XRCC4 DNA repair genes in systemic lupus erythematosus Lupus 2008 17 988 995 10.1177/0961203308093461 18852222

[B22] Fernandez D Perl A Metabolic control of T cell activation and death in SLE Autoimmun Rev 2009 8 184 189 10.1016/j.autrev.2008.07.041 18722557 PMC2680195

[B23] Gergely P Jr Grossman C Niland B Puskas F Neupane H Allam F Banki K Phillips PE Perl A Mitochondrial hyperpolarization and ATP depletion in patients with systemic lupus erythematosus Arthritis Rheum 2002 46 175 190 10.1002/1529-0131(200201)46:1<175::AID-ART10015>3.0.CO;2-H 11817589 PMC4020417

[B24] Fan L Fuss JO Cheng QJ Arvai AS Hammel M Roberts VA Cooper PK Tainer JA XPD helicase structures and activities: insights into the cancer and aging phenotypes from XPD mutations Cell 2008 133 789 800 10.1016/j.cell.2008.04.030 18510924 PMC3055247

[B25] Olichon A Guillou E Delettre C Landes T Arnaune-Pelloquin L Emorine LJ Mils V Daloyau M Hamel C Amati-Bonneau P Bonneau D Reynier P Lenaers G Belenguer P Mitochondrial dynamics and disease, OPA1 Biochim Biophys Acta 2006 1763 500 509 10.1016/j.bbamcr.2006.04.003 16737747

[B26] Scarpulla RC Nuclear activators and coactivators in mammalian mitochondrial biogenesis Biochim Biophys Acta 2002 1576 1 14 12031478 10.1016/s0167-4781(02)00343-3

[B27] Nagy G Barcza M Gonchoroff N Phillips PE Perl A Nitric oxide-dependent mitochondrial biogenesis generates Ca^2+ ^signaling profile of lupus T cells J Immunol 2004 173 3676 3683 15356113 10.4049/jimmunol.173.6.3676 PMC4034140

[B28] Ishikawa S Mima T Aoki C Yoshio-Hoshino N Adachi Y Imagawa T Mori M Tomiita M Iwata N Murata T Miyoshi M Takei S Aihara Y Yokota S Matsubara K Nishimoto N Abnormal expression of the genes involved in cytokine networks and mitochondrial function in systemic juvenile idiopathic arthritis identified by DNA microarray analysis Ann Rheum Dis 2009 68 264 272 10.1136/ard.2007.079533 18388159

[B29] Salvioli S Capri M Valensin S Tieri P Monti D Ottaviani E Franceschi C Inflamm-aging, cytokines and aging: state of the art, new hypotheses on the role of mitochondria and new perspectives from systems biology Curr Pharm Des 2006 12 3161 3171 10.2174/138161206777947470 16918441

[B30] McInnes IB Schett G Cytokines in the pathogenesis of rheumatoid arthritis Nat Rev Immunol 2007 7 429 442 10.1038/nri2094 17525752

[B31] Yang D Elner SG Bian ZM Till GO Petty HR Elner VM Pro-inflammatory cytokines increase reactive oxygen species through mitochondria and NADPH oxidase in cultured RPE cells Exp Eye Res 2007 85 462 472 10.1016/j.exer.2007.06.013 17765224 PMC2094037

[B32] Holohan C Szegezdi E Ritter T O'Brien T Samali A Cytokine-induced β-cell apoptosis is NO-dependent, mitochondria-mediated and inhibited by BCL-XL J Cell Mol Med 2008 12 591 606 10.1111/j.1582-4934.2007.00191.x 18081694 PMC3822546

[B33] Autret A Martin SJ Emerging role for members of the Bcl-2 family in mitochondrial morphogenesis Mol Cell 2009 36 355 363 10.1016/j.molcel.2009.10.011 19917245

